# *Escherichia coli* Cytotoxic Necrotizing Factor 1 (CNF1): Toxin Biology, *in Vivo* Applications and Therapeutic Potential 

**DOI:** 10.3390/toxins2020283

**Published:** 2010-02-23

**Authors:** Alessia Fabbri, Sara Travaglione, Carla Fiorentini

**Affiliations:** Department of Therapeutic Research and Medicines Evaluation, Istituto Superiore di Sanità, Viale Regina Elena 299, 00161 Roma, Italy; Email: sara.travaglione@iss.it (S.T.); carla.fiorentini@iss.it (C.F.)

**Keywords:** CNF1, *Escherichia coli*, memory, vaccine adjuvant, drug delivery agent, pain

## Abstract

CNF1 is a protein toxin produced by certain pathogenic strains of *Escherichia coli*. It permanently activates the regulatory Rho, Rac, and Cdc42 GTPases in eukaryotic cells, by deamidation of a glutamine residue. This modification promotes new activities in cells, such as gene transcription, cell proliferation and survival. Since the Rho GTPases play a pivotal role also in several processes *in vivo*, the potentiality of CNF1 to act as a new pharmacological tool has been explored in experimental animals and in diverse pathological contexts. In this review, we give an update overview on the potential *in vivo* applications of CNF1.

## 1. Introduction

Bacterial protein toxins, the principal virulence factors of pathogenic bacteria, represent one of the main bacterial strategies to interact with mammalian cells. Toxins are extremely fascinating molecules. By diverse and sophisticated mechanisms, they manipulate the host cell functions in a way that can favor the survival and spreading of the microbes. On the other hand, although detrimental to the susceptible host during an infection, the activities of several bacterial toxins can be exploited for medical applications. It is, in fact, of paramount importance to study how a bacterial toxin acts at molecular level, not only to contrast bacterial diseases, thus subverting its harmful effect but also to utilize the toxin as an instrument to control certain cellular pathways [1].

Toxins are the product of a long-term evolution, and can attack crucial events in the vital processes of living organisms, such as components of the protein synthesis machinery, actin polymerization, signal transduction pathways, intracellular trafficking of vesicles as well as immune and/or inflammatory responses. For this reason, in the last years, some of them, besides representing valuable tools for analysis of cellular physiology, have also been successfully employed for treatment or prevention of human diseases. The most renowned example is botulinum toxin that, despite its deadly toxic effect, it is now widely used in therapy in very small doses to treat different muscle disorders, such as muscle spasms, or in various settings for cosmetic procedures [2].

Here we deal with the Cytotoxic Necrotizing Factor 1 (CNF1) from *Escherichia coli* that exerts a specific enzymatic activity on pivotal molecular switches in eukaryotic cells. In recent years, due to its specificity of action on the Rho GTPases (see below), CNF1 has been proved, in experimental animals, to possess vaccine adjuvanticity properties, to be effective in enhancing learning and memory, and to induce analgesia. 

## 2. The Cytotoxic Necrotizing Factor 1: *In Vitro* Effects

CNF1 is a bacterial protein toxin produced by certain pathogenic strains of *E. coli* [3] occasionally detected in isolates from feces of children with diarrhea [4,5,6] but, more frequently, responsible of extra-intestinal infections, particularly in the urinary tract (UTIs) [3,7,8]. Also, these strains can be detected in cases of bacteriaemia [9] and of meningitis in neonates [10]. 

CNF1 belongs to the so-called dermonecrotic toxins family, including CNF1, CNF2 from *E. coli*, CNFY from *Yersinia pseudotubercolosis*, and the dermonecrotic toxin DNT from *Bordetella*, all sharing considerable functional and amino acid homologies [11].

### 2.1. CNF1 structure and endocytosis

CNF1, first described in 1983 by Caprioli and coworkers as a toxin capable of causing multinucleation ("cytotoxic") in cultured cells and necrosis in rabbit skin ("necrotizing") [12,13], is a 1,014 amino acid single-chain multidomain protein, containing a N-terminal receptor-binding domain and a C-terminal catalytic domain, which modifies a specific cellular target in the host cell cytosol [14,15]. The two domains are separated by an amino-acidic region consisting of two short membrane spanning helices H1 (350-372) and H2 (387-412), which are involved in membrane translocation [16].

CNF1 binds to the surface of cultured epithelial cells with high affinity [17]. The domain that mediates the interaction with the cell surface (amino acids 53 to 190) is able to inhibit the holotoxin activity when given to cells in excess [18]. Using this receptor binding domain as a bait in the yeast two hybrid system, the ubiquitously expressed 37 kDa precursor of the 67 kDa laminin receptor (LRP) could be identified as an interaction partner for CNF1 [19]. Indeed, RhoA activation and bacterial uptake (which are the consequences of CNF1 enzymatic activity in cells, see below) are inhibited by exogenous LRP or LRP antisense oligodeoxynucleotides [19]. Moreover, recently, Kim and collaborators have demonstrated that an *E. coli* K1 strain that expresses CNF1 can be internalized by HBMECs after the toxin binds to the mature laminin receptor [20]. Both the precursor and mature forms of the laminin receptor are expressed on the surface of eukaryotic cells and are therefore accessible to CNF1 released from bacteria; however, it is unclear whether CNF1 binds preferentially to one of the two receptor forms.

However, competition studies with CNF1 and CNFY suggest that CNF1 has a co-receptor in addition to the laminin receptor. Inhibition of heparansulfate proteoglycan (HSPG) expression causes delayed cell entry of CNF1 and no uptake of CNFY [21]. Thus, HSPGs may represent the receptor for CNFY and the co-receptor for CNF1, respectively. Furthermore, a recent study also suggests a co-receptor for CNF1 due to a second receptor binding part within the CNF1 sequence including the amino acids 683 to 730 [22].

After binding to its receptor, CNF1 enters endocytic vesicles by receptor-mediated endocytosis, which is independent of clathrin and of sphingolipid-cholesterol-rich membrane microdomains (lipid rafts), including caveolae [17]. Once inside vesicles, the toxin is routed to the endosomal compartment [17], where its catalytic domain is transferred into the cytosol. It was postulated that the hydrophilic loop, which separates H1 and H2 helices present in the middle part of the CNF1 molecule, is essential for translocation of the toxin into the host cell cytosol [16].

Very recently, it has been demonstrated that an approximately 55-kDa fragment of CNF1, which contains the catalytic domain and an additional part of the toxin, is present in the cytosol [23]. The processing of this fragment requires an acidic pH and insertion of the toxin into the endosomal membrane. This is in line with the fact that the pH-dependent membrane translocation step of CNF1 could be mimicked at the level of the plasma membrane by a brief exposure to a pH of ≤5.2 [17]. The cleavage site of CNF1 is located between amino acids 536 and 542. Experiments with CNF1 mutants, which are deficient in the cleavage site, clearly show that the processing and release of the catalytic part from the endosomes is essential for the full biologic activity. It is likely that this release occurs from late endosomes, because the destruction of the microtubules, which are essential for the maturation from early to late endosomes, results in weaker CNF1 toxicity [17,21].

### 2.2. CNF1 enzymatic activity

The C-terminal part of CNF1 (amino acids 720 to 1014) harbors the full catalytic activity [24,25]. Cysteine 866 and histidine 881 have been found to be crucial for the catalytic activity of CNF1 [25]. The crystal structure identified the third residue of the catalytic triad, valine 833 [14].

**Figure 1:**
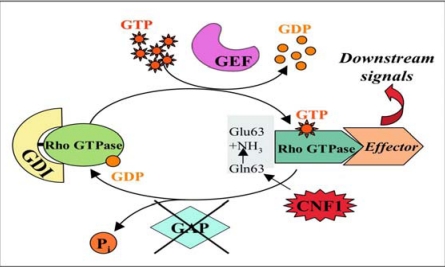
**Rho GTPases activation/deactivation cycle.** Rho GTPases are molecular switches that cycle between an inactive GDP-bound form and an active GTP-bound one. Activation of Rho GTPases occurs by stimulation with a guanine exchange factor (GEF) that causes the release of GDP and the binding of GTP. In the GTP-bound form, Rho proteins interact with effector molecules initiating a downstream response. To exert their activity Rho proteins require to be attached to membranes. As soon as the activated GTPase has initiated the cellular response, a GTPase activating protein (GAP), by hydrolyzing GTP into GDP turns back these proteins to their GDP-bound state, to complete the cycle and terminate the signal transduction. The conformational changes, induced by the binding of GTP or GDP, occur inside two molecular domains of the Rho proteins called *switches* that are responsible of the coupling of the G-proteins with their effectors (*switch 1*) or allow the interaction of the activated GTPase with GAPs (*switch 2*). Besides GEF and GAP proteins, a third factor that regulates Rho GTPases activity is the guanine dissociation inhibitor (GDI), which maintains Rho in the cytoplasm in the inactive form (linked to GDP). The enzymatic activity of CNF1 consists in the deamidation of a specific glutamine residue, located in the *switch 2* domain, into glutamic acid thus impairing the role of GAP and permanently blocking Rho proteins in their activated GTP-bound state.

The cytoplasmic target of CNF1 is represented by Rho GTP-binding proteins, important molecular switches belonging to the Ras superfamily that oscillate between an inactive GDP-bound form [26] and an active GTP-bound form, which activates effector proteins [27]. Transitions between the two forms are primarily under the control of regulatory proteins (Figure 1) [28,29]. Rho proteins possess a low intrinsic GTPase activity and a strong GTPase activity stimulated by the regulatory protein GAP. The conformational changes, induced by the binding of GTP or GDP, occur inside two molecular domains of the Rho proteins called *switches* that are responsible of the coupling of the G-proteins with their downstream effectors (*switch 1*) or allow the interaction of the activated GTPase with the regulatory proteins GAPs (*switch 2*). 

The enzymatic activity of CNF1 consists in the deamidation of a specific glutamine residue located in the *switch 2* domain of the G proteins (glutamine 63 of Rho [30,31] or glutamine 61 of Rac and Cdc42 [32], which is pivotal for the GTPase activity of Rho proteins, either intrinsic or stimulated by GAP [33]. CNF1 modifies glutamine into glutamic acid, thus impairing the role of GAP and permanently blocking Rho proteins in their activated GTP-bound state. This causes constitutive activity of these proteins on their effectors (Figure 1).

The threshold of activation of Rho proteins by CNF1 is, however, attenuated because of a concomitant decrease of their cellular levels, due to the depletion of activated-Rho GTPases by ubiquitin-mediated proteasomal degradation [34]. 

It is worth noting that the activity of CNF1, with its ability to switch on the Rho GTPases and then coerce their degradation in the proteasome, shows similarities to the transient activation of Rac and Cdc42 proteins by pathogenic *Salmonella* bearing the virulence factors SopE and SptP, two Rac/Cdc42 GEF (activation) and GAP (inhibition) functional analogs, respectively [35]. This bacterium first activates the Rho GTPases, by the GEF-like toxin SopE, to promote engulfment of bacteria into cells and soon after, once inside, deactivates the GTPases *via* a GAP-mimicking protein (SptP), thus allowing a moderate threshold of Rho protein activation for a high invasion efficiency [36].

### 2.3. Effects of CNF1 on eukaryotic cultured cells

Rho GTPases regulate the organization and dynamic of the actin cytoskeleton through their interplay with specific effector proteins [27,37].

Indeed, by activating Rho proteins, CNF1 provokes a remarkable reorganization of the actin cytoskeleton in human epithelial cells that consists in the assembly of F-actin in prominent stress fibers, membrane ruffles and filopodia [38,39]. Besides extreme flattening of the cell body, CNF1-treated cultured cells acquire a multinucleated phenotype. This seems to arise from inhibited cytokinesis with ongoing nuclear division [23].

The extensive modifications of the microfilament system induced by the toxin provokes a potent and aspecific phagocytic behavior, epithelial cells acquiring the ability to capture and internalize different types of particles, such latex beads, apoptotic cells and also bacteria [40,41], through an endocytic mechanism known as macropinocytosis. As above described, CNF1-activated Rho GTPases undergo sensitization to ubiquitylation and subsequent proteosomal degradation [34], a process that would turn off the ruffling process, thus allowing an efficient internalization of bacteria inside the cells. Since degradation of Rac increases cell motility and leads to enhanced internalization of bacteria, it was speculated that sequential Rho GTPase activation and inactivation limits the inflammatory response and promotes survival of bacteria [34].

Although Rho, Rac and Cdc42 mainly regulate the actin cytoskeleton organization [42], it is known that these proteins are also involved in many other cellular processes. Thus, the consequence of CNF1-induced Rho activation is the induction of a number of actin-dependent or -independent phenomena, such the induction of macropinocytosis (see above), contractility, cell spreading [38], assembly of focal adhesion plaques [43], transcription [44], cell cycle progression [45], as well as the ability to manipulate cell differentiation [46]. 

As concerns DNA transcription, we reported that CNF1 can activate the nuclear factor-κB (NF-κB) [44] in epithelial cells. Such an activation is responsible for the ability of the toxin to stimulate the expression of pro-inflammatory factors [47] and to protect host cell from apoptotic stimuli [48,49,50]. Hence, CNF1 is not cytotoxic, as suggested by its name, but rather can be considered as a pro-survival factor. Indeed, we have shown that CNF1 hinders UVB-induced apoptotic cell death in HEp-2 epithelial cells. In particular, the toxin protects cells from the experimentally-induced rounding up and detachment and improves cell spreading and the ability of cells to adhere to each other and to the extracellular matrix by modulating the expression of proteins related to cell adhesion (integrins, focal adhesion kinase, cadherins, catenins) [48]. Moreover, CNF1 protects epithelial cells against the drop of the mitochondrial membrane potential provoked by UVB radiation and increases the expression of the anti-apoptotic members of the Bcl-2 family, Bcl-2 and Bcl-X_L_[49]. In particular, CNF1 up-regulates the anti-apoptotic protein Bcl-2 through the activation of the Rac1/PI3K/Akt/IKK/NF-κB pro-survival pathway. This, in turn, leads to a remarkable modification in the architecture of the mitochondrial network, mainly consisting in the appearance of elongated and interconnected mitochondria, which contributes to the pro-survival activity of the toxin [50].

As above mentioned, CNF1-induced NF-κB activation ensues the transcription and release of pro-inflammatory cytokines, such as IL-6, IL-8 and TNF-α in uroepithelial [47] and endothelial [51] cells and up-regulates the transcription of cyclooxygenase- 2 (COX-2) [52]. Moreover, after cell activation by CNF1, epithelial cells show an increase in oxygen consumption and generate superoxide anions [53].

## 3. The Cytotoxic Necrotizing Factor 1: *In Vivo* Effects

Understanding how CNF1 acts at the molecular level is of importance for using the toxin as an instrument to control certain cellular pathways, thus converting a detrimental toxin effect into a favorable one. Rho family GTPases are involved in various events being powerful cellular regulators and thus rendering CNF1 an attractive putative “drug” in different therapeutic purposes.

### 3.1. Learning and memory

Memory formation is thought to involve the rearrangement of synaptic connections in neuronal networks and this is governed by the neuronal actin cytoskeleton [54,55]. Actin polymerization is chiefly regulated by small GTPases, and in this context, we have recently reported that constitutive activation of Rho family GTPases, induced by a single intracerebroventricular injection of CNF1 in C57BL/6 and CD1 mice, resulted in rearrangements of actin in cortical neural cells and induced sustained enhancement of cognitive performances [56]. In particular, CNF1 caused a general improvement in associative and spatial learning, as demonstrated in various behavioral tasks. These CNF1-induced effects were persistent for months and were absent in animals injected with CNF1 C866S, the inactive toxin that lacks the enzymatic activity following the substitution of serine to cysteine at position 866.

**Figure 2:**
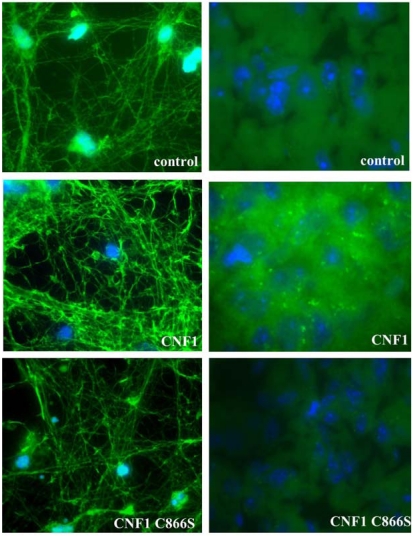
**Effects of CNF1 on actin.** Fluorescence microscopy analysis of primary neuronal cells derived from the mouse cortex (left panels) and cerebral sections of injected mice (right panels). Sections and cells were stained with FITC-phalloidin to detect actin cytoskeleton and with Hoechst 33258 to visualize nuclei. The pictures show that CNF1 is able to induce an enrichment in actin staining both *in vivo* and in cell cultures whereas the mutant toxin devoid of catalytic activity lacks the ability to modify the actin cytoskeleton.

Learning and memory depend on the morphology and dynamics of dendritic spines and their development and structural plasticity is controlled by Rho GTPases and actin cytoskeleton. In line with this, CNF1 by activating Rho proteins, induced an enrichment in actin staining both in cerebral sections of injected mice and in primary neuronal cells derived from the mouse cortex (Figure 2).

In this context, we demonstrated that the activation of Rho proteins by CNF1 provokes an increase in dendritic surface particularly by spine-like neo-formations, accompanied by actin cytoskeleton enrichment. The above changes were not observed when cells were treated with the mutant CNF1 C866S [56].

Overall, the results suggest that learning ability can be improved through pharmacological manipulation of neural connectivity. These findings raise the possibility that pharmacological agents could be used to enhance the changes in neuronal connectivity associated with memory formation, and the cytoskeletal components are identified as possible targets for such agents. CNF1 can thus be regarded as endowed with long-lasting cognition enhancing properties. 

Furthermore, as well as improving cognition in healthy patients, these drugs could, in theory, be used to alleviate the cognitive impairments exhibited by patients with conditions such as Alzheimer’s Disease.

### 3.2. CNF1 as adjuvant or drug delivery agent

Several bacterial toxins, as for example the cholera toxin, have already been described to be effective adjuvant [57,58]. Adjuvants are vaccine additives that enhance the elicited levels of antibodies and T lymphocyte responses [59] in ways that presumably mimic natural infection. 

Initial studies have demonstrated that CNF1 induces production and secretion of pro-inflammatory and immunomodulatory cytokines *via* the activation of Rho proteins [47,60]. The subsequent investigation of the immunomodulatory effects of CNF1 using a mouse model of oral administration, showed that, similarly to cholera toxin, CNF1 elicited adjuvanticity in anti-OVA responses, both systemic and mucosal [60]. In fact, animals orally immunized with OVA, a prototype soluble antigen, and co-administered with CNF1 displayed serum IgG anti-OVA responses. The immunoadjuvant properties of CNF1 are related to its enzymatic activity on Rho proteins. In fact, the catalytic inactive CNF1 C866S failed to enhance serum anti-OVA responses and the catalytic domain of the closely related *Bordetella* dermonecrotic toxin (DNT), a Rho activating toxin, also showed immunoadjuvant properties. Thus, the host response to CNF1, in addition to promoting innate immune effectors and regulators, may also stimulate the adaptative immune system. 

In the same field, some years later, CNF1 was reported to act as a mucosal adjuvant in immunization against tetanus toxoid through nasal route [61]. CNF1 *plus* tetanus toxoid induced a specific production of anti-Tetanus toxin (TeNT) IgG that was able to exert a protective effect when mice were challenged with TeNT. Conversely, mice immunized with the tetanus toxoid *plus* the mutant CNF1 C866S died when challenged with TeNT, consistently with the lack of anti-TeNT IgG response. An analysis of the TeNT-specific IgG subclasses revealed that the serum antigen response was predominantly of IgG1 type, followed by IgG2a whereas seric IgA were found but at a lesser extent. This response remained unchanged two and six months later indicating a long lasting specific anti-TeNT response induced by CNF1.

Manipulation of Rho proteins thus provides a possible new approach for the development of effective mucosal immunoadjuvants.

### 3.3. Analgesic activity of CNF1

Inflammatory pain is controlled by a complex framework of molecular events, either at the cell surface or intracellularly, which have only partially been elucidated. In particular, it seems that the actin cytoskeleton influences pain-related signaling [62,63,64]. 

The formalin pain model is a well-established and used model to assess animals’ behavioral responses to persistent pain and to examine analgesic effects of drugs. Formalin induced pain derives from prolonged tissue damage and represents a model for pain states encountered clinically, such as post-operative pain [65]. In this context, we have recently reported that peripheral or central administration of CNF1 potently counteracted formalin-induced inflammatory pain in mice [66]. The two phases characterizing the formalin-induced pain, were affected by CNF1 in a different way, depending on the route of administration. In fact, i.c.v. injection of the toxin was able to affect both phases indicating a central modulating action of CNF1 on neuronal processes associated with both the acute phase and the inflammatory tonic phase of the formalin-induced pain. On the other hand, peripherally injected CNF1 influenced only the second phase. 

The effects of CNF1 on inflammatory pain were associated with sustained Rac activation and the consequent actin cytoskeleton rearrangement. The toxin involves the activation of the Akt/IKK/NF-κB pathway, as reported in cultured cells, and increases the expression of μ-opioid receptors (MORs) in particular in the PAG area, the part of the pain-modulating circuits characterized by a high density of MORs [67]. Thus, CNF1, starting from Rac activation, could switch on a series of intracellular events including MORs upregulation which probably leads to the observed analgesic effects. This is in line with the fact that CNF1 was unable to reduce pain perception in knockout mice for MORs (μ-/-) despite the occurrence of cerebral Rac activation. The hypothesis is that CNF1-induced MORs upregulation could provide additional links to the endogenous opioids released after formalin stimulation, thus reducing the pain threshold. This finding discloses new possibilities for the pharmacological control of inflammatory pain. 

## 4. Conclusions

Bacterial toxins play an important role in infectious diseases, and several of them are amongst the most potent biological agents known to man. Cholera, pertussis, botulinum, clostridium and tetanus toxins are all produced by bacteria and, in many cases, it is the toxin produced and not the infectious agent itself that causes pathology. Also, botulinum, anthrax, and other toxins, have potentially devastating effects if misused as an agent of biological terror. However, although detrimental to the susceptible host during an infection, the activities of several bacterial toxins can be exploited for medical applications [1].

In this review, we have focused our interest on CNF1, on its applications and therapeutical potential, describing the state of art of its possible employment in therapy. Although the first report on this aspect concerns the adjuvancy of CNF1 and its ability to engage the immune system, in our view, the most fascinating aspect of this toxin resides in its potentiality to act on the “synaptic plasticity” in the nervous system. Synaptic plasticity is acknowledged to depend on actin dynamics that are controlled by Rho GTPases. Importantly, Rho GTPase signaling and spine morphology are consistently affected in human memory disorders, including Alzheimer’s Disease (AD). Therefore, CNF1 acts on a mechanism that might represent a rate-limiting step for cognition in humans. Memory disorders are characterized also by alterations in NF-κB signaling, which lies downstream Rho GTPases and has been implicated in adult neurogenesis modulation. With this in mind, it stems the uniqueness of CNF1 that controls actin dynamics *via* Rho signaling as well as NF-κB activation in any kind of tissue investigated so far, including the brain. Hence, a pharmacological intervention at the level of the Rho GTPases themselves or their downstream effectors (NF-κB) may be the most effective strategy to manipulate synaptic plasticity underlying memory. These considerations imply the potential pharmacological importance of the molecule which could, in theory, be used to alleviate the impairments exhibited by patients with cognitive disorders.

On the other hand, Rho proteins, particularly Rac, are also involved in inflammatory pain perception. The ability to activate the Rac GTPase at both cerebral and peripheric sites, renders CNF1 a useful tool for the comprehension of the fine mechanisms underlying pain. More importantly, these findings can disclose a new scenario for the pharmacological control of inflammatory pain, that is the possibility to overcome the “classical” receptor ligation-mediated pain control by introducing a new, intracellularly regulated path aimed at the modulation of pain perception. 

Although it has been shown that the i.c.v injection of the entire CNF1 toxin causes no lethal effects in animals [56,66], it still remains difficult to propose the use of such a high molecular weight protein (114 kDa) for therapeutic purposes. This problem could be overcome by constructing recombinant molecules composed by CNF1-derived peptides that maintain the catalytic Rho GTPase activating capacity and that will be vehiculated in the CNS with delivery systems such as nanoparticles. 

In conclusion, the presumed therapeutic properties of CNF1 arises a question: can we consider this toxin as a “magic bullet” for the nervous system?
